# Thoracoscopic surgery for atrial fibrillation in a patient with persistent left superior vena cava: a case report

**DOI:** 10.1186/s44215-023-00079-6

**Published:** 2023-08-01

**Authors:** Go Seimei, Takahashi Shinya, Ohtsuka Toshiya, Takasaki Taiichi

**Affiliations:** 1 Department of Cardiovascular Surgery, Hiroshima University Hospital, Kasumi 1-2-3, Minamiku Hiroshima-City, Hiroshima Japan; 2 Department of Cardiovascular Surgery, NewHeart Watanabe Institute, Tokyo, Japan

**Keywords:** PLSVC, Stapler closure of LAA, PV isolation

## Abstract

**Background:**

A persistent left superior vena cava (PLSVC) is an anomaly of the thoracic venous system that flows into the right atrium via an enlarged coronary sinus. When performing pulmonary vein isolation and left atrial appendage closure, the PLSVC can interfere with the procedure. We have performed thoracoscopic surgery on such a patient and would like to share our experience.

**Case presentation:**

The patient is a 70-year-old male with a 1-year history of repeated tachycardia with chest discomfort due to paroxysmal atrial fibrillation. Contrast-enhanced computed tomography revealed the presence of a PLSVC that flows into the right atrium. The hepatic vein flows directly into the right atrium, whereas the inferior vena cava enters the thoracic cavity next to the descending aorta and flows into the parazygous vein and PLSVC. We performed thoracoscopic stapler closure of the left atrial appendage and epicardial clamp-isolation of the pulmonary veins and the PLSVC. There was concern that the PLSVC would interfere with the visual field needed to perform the procedure. We carefully removed the adhesions to the surrounding tissue and provided mobility to the PLSVC and expanded it ventrally. As a result, the procedure could be performed safely and without complications.

**Conclusions:**

Our results demonstrate that in cases where catheter ablation is difficult for anatomical reasons, thoracoscopic stapler closure of the LAA and epicardial clamp-isolation of pulmonary veins may be a viable option.

## Background

A persistent left superior vena cava (PLSVC) is an anomaly of the thoracic venous system that flows into the right atrium via an enlarged coronary sinus. When performing pulmonary vein isolation and left atrial appendage closure, the PLSVC can interfere with the procedure. In this case, we performed thoracoscopic stapler closure of the left atrial appendage (LAA) and epicardial clamp-isolation of pulmonary veins in a patient with visceral retroversion below the diaphragm and PLSVC.

## Case presentation

**Fig. 1:**
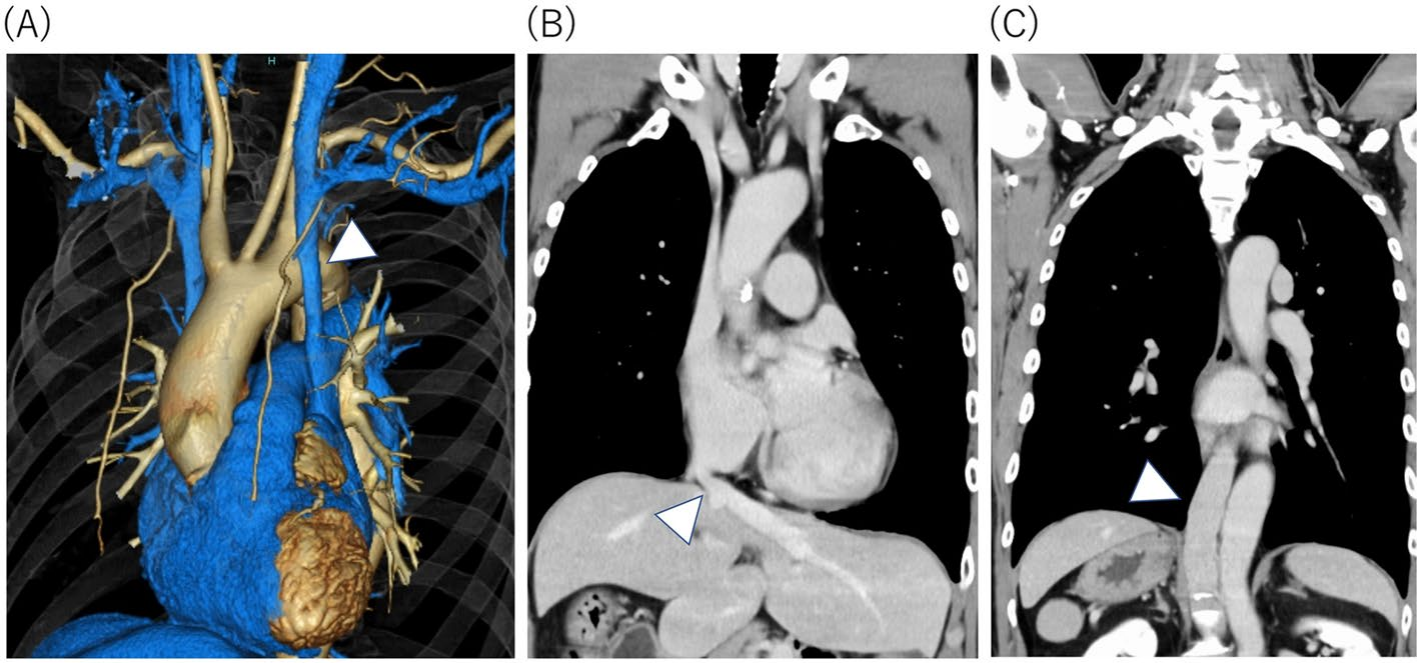
**(A)** Contrast-enhanced computed tomography showed the presence of the PLSVC (triangular arrow) that flows into the right atrium. **(B)** The hepatic vein (triangular arrow) flows directly into the right atrium. **(C)** The inferior vena cava (triangular arrow) enters the thoracic cavity next to the descending aorta and flows into the parazygous vein and PLSVC

The patient is a 70-year-old male with a 1-year history of repeated tachycardia with chest discomfort due to paroxysmal atrial fibrillation. He was referred to our cardiology department for treatment. Contrast-enhanced computed tomography revealed the presence of a PLSVC that flows into the right atrium (Fig. 1(A)). The hepatic vein flows directly into the right atrium (Fig. 1(B)), whereas the inferior vena cava enters the thoracic cavity next to the descending aorta and flows into the parazygous vein and PLSVC (Fig. 1(C)). Abdominal organs such as the liver, spleen, and stomach were viscerally retroverted. Because of these anatomical anomalies, it was determined that endocardial ablation by catheters would be difficult. As the patient wanted aggressive treatment instead of drug therapy, he was referred to our department for surgery.

**Fig. 2:**
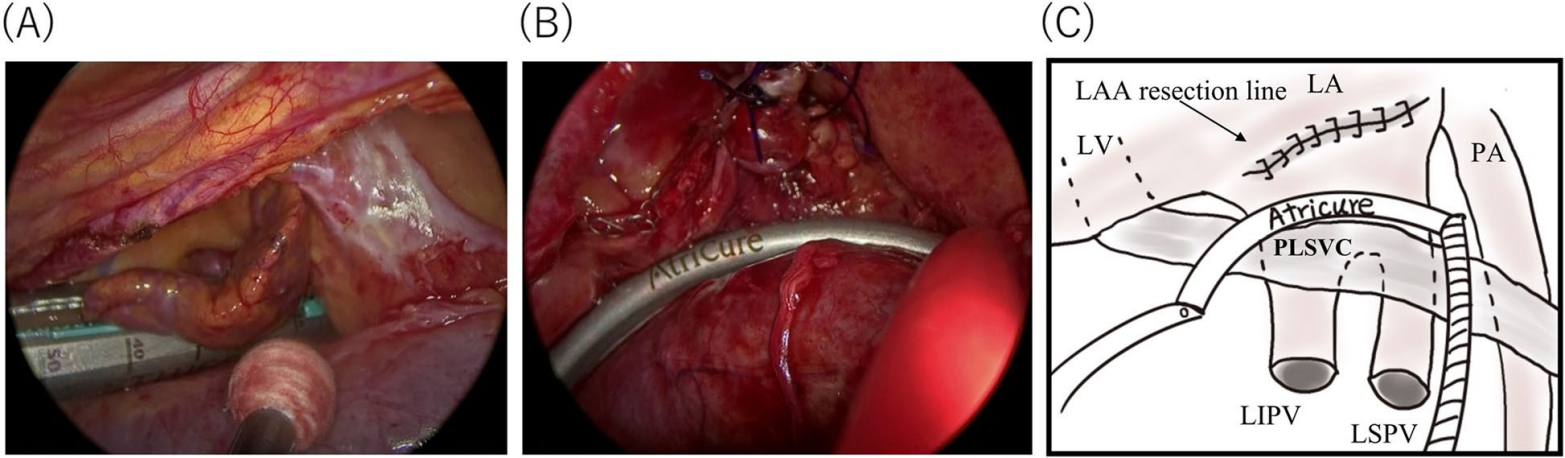
**(A)** After ensuring that the root of the LAA was fully exposed, the LAA was closed with an automatic cut-and-staple device. **(B)** The PLSVC was entered into the clamp and bitted with the clamp device with the left PVs, and then the PV isolation was conducted. **(C)** This illustrates **(B)**

Under general anesthesia via a double-lumen endotracheal tube that allows for a hemi pulmonary collapse, the patient was placed in the supine position with his hands pulled down behind his back. Transesophageal echocardiography confirmed that there were no clots in the LAA that could cause procedure-related thromboembolism. Thoracoscopic procedures were completed through four ports in each side. The pericardium was incised on its dorsal side with attention to the transverse nerve. When the pericardiotomy was expanded ventrally, a thick PLSVC was observed in the front of the visual field. The PLSVC was gently lifted to avoid damaging it, and the dissection was performed cephalad. An adhesion was found between the left pulmonary artery and the PLSVC. The degree of adhesion was moderate, which could not be removed with blunt detachment and required an electrocautery scalpel to remove it. After ensuring that the root of the LAA was fully exposed, the LAA was closed with an automatic cut-and-staple device (ECHELON FLEX Stapler 60 mm, ETHICON, USA) (Fig. 2(A)). The endoscope revealed a partial residual LAA on the cephalic side, which was closed by tightening the residual with a device (Endloop PDS2, ETHICON, USA). Next, the left pulmonary vein (PV) was taped, and a radiofrequency bipolar epicardial coagulator (Isolator Synergy Clamps, Atricure, USA) was inserted from the caudal side toward the cephalic side. The PLSVC was entered into the clamp and bitted with the clamp device with the left PVs, and then the PV isolation was conducted (Fig. 2(B)). Next, we moved to the right side to isolate the right PVs with the same method. The operation was uneventfully completed in 145 min. Warfarin was resumed the day after the operation and is planned to discontinue 1 month later. The patient did well postoperatively and had no atrial fibrillation and was discharged from our hospital 6 days after the operation. This patient is currently being monitored as an outpatient and is doing well with no recurrence of atrial fibrillation.

## Discussion and conclusions

According to the guidelines of the Japanese Cardiology Society, catheter ablation therapy for drug-resistant paroxysmal atrial fibrillation is class 1a [1]. However, catheter ablation treatment was difficult in this case due to the presence of a PLSVC, visceral retroversion, and abnormal venous run. Nevertheless, as the patient strongly desired a therapeutic procedure, we chose a surgical procedure. To minimize the invasiveness, we selected a port access thoracoscopic procedure developed from a mini thoracotomy procedure invented by Dr. Wolf and Dr. Ohtsuka [2, 3]. This method has been reported to have good mid-term outcomes [4]. There was concern that the PLSVC would interfere with the visual field needed to perform the procedure. We carefully removed the adhesions to the surrounding tissue and provided mobility to the PLSVC and expanded it. As a result, the procedure could be performed safely. Dr. Gao and colleagues reported that ablation aiming for PLSVC isolation is required in most patients because recurrence of atrial arrhythmia after catheter ablation is relatively common [5]. In this case, the PLSVC was ablated together because it ran close to the left PVs and was in a position to be clamped simultaneously by the ablation device.

Surgical treatment of atrial fibrillation in patients with PLSVC, visceral retroversion, and venous malrotation is uncommon. We performed an operation on a patient with these anatomical anomalies safely. Our results demonstrate that in cases where catheter ablation is difficult for anatomical reasons, the thoracoscopic stapler closure of the LAA and epicardial clamp-isolation of pulmonary veins may be a viable option.

## Data Availability

There are no additional data to disclose.
